# Impulsive fractional order integrodifferential equation via fractional operators

**DOI:** 10.1371/journal.pone.0282665

**Published:** 2023-03-16

**Authors:** Ahmad Al-Omari, Hanan Al-Saadi

**Affiliations:** 1 Al al- Bayt University, Department of Mathematics, Faculty of Sciences, Mafraq, Jordan; 2 Umm Al-Qura University, Department of Mathematics, Faculty of Applied Sciences, Makkah, Saudi Arabia; University of Porto Faculty of Engineering: Universidade do Porto Faculdade de Engenharia, PORTUGAL

## Abstract

In this paper, we establish the existence and uniqueness of the solution to fractional equations abstract integrodifferential equation with impulsive as

$$\begin{array}{ccc} \frac{d^{\eta }w\left(z\right)}{dz^{\eta }}+\zeta w\left(z\right) & = & f\left(z,w\left(z\right)\right)+K\left(w\right)\left(z\right),\;z>z_{0},\;z\neq z_{k},\;0<\eta \leq 1,\\ {\Delta w|}_{z=z_{k}} & = & I_{k}\left(w\left(z_{k}^{-}\right)\right),\;k=1,...,m,\\ w\left(z_{0}\right)+\varphi \left(w\right) & = & w_{0}\in X, \end{array}$$

where

$$\begin{array}{c} K\left(w\right)\left(z\right)=\int_{z_{0}}^{z}q\left(z-s\right)\;g\left(s,w\left(s\right)\right)\;ds, \end{array}$$

and we used the fixed point theorems due to Banach space. The main conclusion is obtained by using fractional calculus, operator semigroup and fixed point theorem. Finally, in support, an example is presented to validate the obtained results.

## 1 Introduction

Many authors interested in fractional differential equations for many decades. In [1], Hsiang and Chang proved the existence of mild and classical solutions of nonlocal Cauchy problems. Also, show that the linear part generates an analytic compact bounded semigroup, and that the nonlinear part is a Hölder continuous function with respect to the fractional power norm of the linear part. In [2], Mahdy gave a new numerical method for solving a linear system of fractional integrodifferential equations. He conceder the fractional derivative is considered in the Caputo sense to demonstrate the accuracy and applicability of the presented method. In [3], Aly Abdou proposed a new fractional sub-equation method with a fractional complex transform for constructing exact solutions of fractional partial differential equations arising in plasma physics in order to a modified Riemann-Liouville derivative. In [4], N’Guerekata discussed the existence and uniqueness of solutions to the Cauchy problem for the fractional differential equation with non-local conditions as.

$$D^{q}x\left(t\right)=f\left(t,x\left(t\right)\right),\;t\in \left[0,T\right],\;x\left(0\right)+g\left(x\right)=x_{0},\;\;\;\text{where}\;\;\;0<q<1,\text{in}\;\text{a}\;\text{Banach}\;\text{space.}$$

In [5], Mophou et al. order to the existence solution of the Cauchy problem for the fractional differential equation with non local conditions in *R*^*n*^:

$$D^{q}x\left(t\right)=Ax\left(t\right)+f\left(t,x\left(t\right)\right),\;t\in \left[0,T\right],\;x\left(0\right)+g\left(x\right)=x_{0},\;\;\;\text{where},\;\;\;0<q<1.$$

In [6], Pei-Luan and Chang-Jin investigated the boundary value problems of fractional order differential equations with non instantaneous impulse. By some fixed-point theorems, the existence solution of mild solution are established. Reference [7], Chang and Ponce established the existence of mild solutions to a multi-term fractional differential equation.

One of the important topics in ocean wave are the characteristics and mechanism of linear and nonlinear structures like solitary waves, cotidal waves, rogue waves, etc. in fluid mediums (including in seas and oceans plasma physics). The moving waves in ocean or shallow water or generally in fluid mediums is very important part in ocean and in science generally. Since the waves can cause disasters, during several decade the scientists study the behaviors of the waves in different aspects. Mathematicians present several linear and nonlinear differential and integrable equations that describe the nonlinear phenomena in shallow water and water in oceans.

Dabas et al. [8], considered a solution for a semilinear problems and proved its existence and uniqueness. Authors in [9, 10] under the assumption of the generator, our discussion focused on the uniqueness and existence of the solution to the following IDE on an analytic semigroup generator *ζ*, and the maps *f*, *g* which are nonlinear with the kernel *q*,

$$\begin{array}{ccc} \frac{dw(z)}{dz}+\zeta w\left(z\right) & = & f\left(z,w\left(z\right)\right)+\int_{0}^{z}q\left(z-s\right)\;g\left(s,w\left(s\right)\right)\;ds,\;z\in \left(0,T\right],\\ w(0) & = & w_{0}. \end{array}$$

Also, [11–22] introduced some property related to existence of mild solutions for fractional impulsive integro-differential equation. In addition, they focus in improving the techniques to reach the optimal solutions that can predict the phenomena of waves in water or ocean very well.

In, this paper we are order to solving the following impulsive fractional order integrodifferential equation denoted by (IDE) with nonlocal conditions via fractional operators in a Banach space denoted by (BS) *X*;

$$\begin{array}{ccc} \frac{d^{\eta }w\left(z\right)}{dz^{\eta }}+\zeta w\left(z\right) & = & f\left(z,w\left(z\right)\right)+K\left(w\right)\left(z\right),\;z>z_{0},\;z\neq z_{k},\;0<\eta \leq 1,\\ {\Delta w|}_{z=z_{k}} & = & I_{k}\left(w\left(z_{k}^{-}\right)\right),\;k=1,...,m.\\ w\left(z_{0}\right)+\varphi \left(w\right) & = & w_{0}\in X, \end{array}$$ (1)

where

$$\begin{array}{c} K\left(w\right)\left(z\right)=\int_{z_{0}}^{z}q\left(z-s\right)\;g\left(s,w\left(s\right)\right)\;ds. \end{array}$$

We let −*ζ* Analyzes a semigroup *G*(*z*), *z* ≥ 0, on a BS *X* on Eq (1), the map *q* is locally integrable on [*z*_0_, ∞) and real valued, we define the nonlinear maps *f* and *g* on [*z*_0_, ∞) × *X* into *X*. The function *φ* is continuous and will be specified later.

The rest of this paper is organized as follows: In Sec. 2, we introduce some definitions and lemmas; In Sec. 3, a set of sufficient condition is required for the local existence of mild solutions for fractional impulsive integrodifferential equation; In Sec. 4, The proof of global existence of a mild solution are stated. In Sec. 5, an example is given to illustrate our main results.

## 2 Preliminaries

Let *X* be BS and let the closure of the interval [*z*_0_, *T*), *z*_0_ < *T* ≤ ∞ be denoted by *I*. Suppose that −*ζ* ∈ *X* be the infinitesimal-generator of an analytic-semigroup (denoted by IGAS) {*G*(*z*), *z* ≥ 0}. We note that as an analytic semigroup −*ζ* is the infinitesimal generator and −(*ζ* + *αI*) is invertible for *α* > 0, then −(*ζ* + *αI*) is analytic and bounded semigroup. In this way, we can reduce the case in which −*ζ* is an IGAS to the case in which is bounded semigroup and the generator is invertible. In a resolvent set, *ρ*(−*ζ*) is the set of all −*ζ* without loss of generality 0 ∈ *ρ*(−*ζ*). Furtermore, the fractional power *ζ*^*α*^ for 0 < *α* < 1, is define as a closed linear operator with inverse *ζ*^−*α*^ on its domain *D*(*ζ*^*α*^). The following are the basic properties *ζ*^*α*^. For semigroup *G*(*z*) there is an *M* ≥ 1 where ‖*G*(*z*)‖ ≤ *M* for all *z*_0_ ≤ *z* ≤ *T*, the following properties will be used:

**Theorem 2.1** [23]

1. *X*_*α*_ = *D*(*ζ*^*α*^) *is a BS with norm* ‖*x*‖_*α*_ = ‖*ζ*^*α*^*x*‖, *for* *x* ∈ *D*(*ζ*^*α*^).
2. *G*(*z*):*X* → *X*_*α*_, *for each* *x* > 0.
3. *ζ*^*α*^*G*(*z*)*x* = *G*(*z*)*ζ*^*α*^*X*, ∀*x* ∈ *D*(*ζ*^*α*^) *and* *z* > 0.
4. *For every* *z* > 0, *ζ*^*α*^*G*(*z*) *is bounded on* *X* *and there exist* *C*_*α*_ > 0 *and* *δ* > 0 *where*
  $$\begin{array}{c} \|\zeta^{\alpha }G\left(z\right)\|\leq C_{\alpha }z^{-\alpha }e^{-\delta z}\leq C_{\alpha }z^{-\alpha }. \end{array}$$
5. *ζ*^−*α*^ *is a linear bounded operator in* *X* *with* *D*(*ζ*^*α*^) = *im*(*ζ*^−*α*^).
6. *If* 0 < *α* ≤ *β*, *then* $D\left(\zeta^{\beta }\right)↪D\left(\zeta^{\alpha }\right)$.

**Remark 2.1** *In* [1] *and by Theorem 2.1 (2), (3), we can see that the restriction* *G*_*α*_(*z*) *of* *G*(*z*) *to* *X*_*α*_ *is exactly the part of* *G*(*z*) *in* *X*_*α*_. *Let* *x* ∈ *X*_*α*_. *Since*

$$\begin{array}{c} {\left\|G(z)x\right\|}_{\alpha }=\|\zeta^{\alpha }G\left(z\right)x\|=\|G\left(z\right)A^{\alpha }x\|\leq \|G(z)\|\|\zeta^{\alpha }x\|=\|G(z)\|{\left\|x\right\|}_{\alpha } \end{array}$$

*and as z decreases to* 0

$$\begin{array}{c} {\left\|G(z)x-x\right\|}_{\alpha }=\|\zeta^{\alpha }G\left(z\right)x-\zeta^{\alpha }x\|=\|G\left(z\right)\zeta^{\alpha }x-\zeta^{\alpha }x\|\to 0, \end{array}$$

*for all* *x* ∈ *X*_*α*_ *it follows that* (*G*(*z*)_*z* ≥ 0_) *is a family of strongly continuous semigroup on* *X*_*α*_ *and* ‖*G*_*α*_(*z*)‖ ≤ ‖*G*(*z*)‖ *for all* *z* ≥ 0.

We define a mild solution to (1) on *I* a function *w* which is continuous where *w*: *I* → *X* satisfying the following integral equation

$$\begin{array}{c} w(z)=G\left(z-z_{0}\right)\left(w_{0}-\varphi \left(w\right)\right)+\sum_{z_{0}<z_{k}<z}G\left(z-z_{k}\right)I_{k}\left(w\left(z_{k}^{-}\right)\right)\\ +\frac{1}{\Gamma (\eta )}\int_{z_{0}}^{z}{\left(z-s\right)}^{\eta -1}G\left(z-s\right)(f(s,w(s))+K(w)(s))\;ds. \end{array}$$

If there exists a *T*_0_, *z*_0_ ≤ *T*_0_ ≤ *T* and a continuous *w* which defined as *w*: *I*_0_ = [*z*_0_, *T*_0_]→*X*, then we say that (1) has a local mild solution *w* to (1) on *I*_0_.

To start the analysis, we need to know some basic properties and definitions of fractional calculus.

**Definition 2.1** [24] *A space* *C*_*μ*_, *and* μ,P∈R *if* *p* > *μ*, *such that* *f*(*x*) = *x*^*p*^*f*_1_(*x*), *where* *f*_1_ ∈ *C*[0, ∞) *and* *f*(*x*), *x* ≥ 0 *be a real function. The space* *C*_*μ*_ *is called to be* $C_{\mu }^{m}$ *if and only if* *f*^(*m*)^ ∈ *C*_*μ*_, m∈N.

**Definition 2.2** [24] *The fractional integral operator Riemann-Liouville of order* 0 ≤ *α*, *of a function* *f* ∈ *C*_*μ*_, *μ* ≥ −1 *is defined as*

$$\begin{array}{c} I^{\alpha }f\left(x\right)=\frac{1}{\Gamma (\alpha )}\int_{0}^{x}{\left(x-z\right)}^{\alpha -1}f\left(z\right)dz,\;\alpha >0,\;x>0,\\ I^{0}f\left(x\right)=f(x). \end{array}$$

A Riemann-Liouville derivative has certain disadvantages when modeled with fractional differential equations to describe some real-world phenomena. Therefore, we will introduce a modified fractional method $D_{*}^{\alpha }$ proposed by M. Caputo.

**Definition 2.3** [25] *The Caputo fractional derivative of* *f*(*x*) *is defined as*

$$\begin{array}{ccc} DI_{x}^{\alpha }f\left(x\right)= & I^{m-\alpha }D^{m}f\left(x\right) & \\ = & \frac{1}{\Gamma (m-\alpha )}\int_{0}^{x}{\left(x-z\right)}^{m-\alpha -1}D^{m}f\left(z\right)dz, & \text{for}\;m-1\leq \alpha <m,m\in N,\\ & & x>0,\;f\in C_{-1}^{m}. \end{array}$$

## 3 Local existence of mild solutions

For problem (1), in this section we show its existence. Let us list the hypothesis for some *α* ∈ (0, 1). To determine whether a unique mild solution exists (1), we require the following assumption on the maps *f* and *g*.

(H1) Let *W* be an open subset of [0, ∞)×*X*_*α*_. The map *f* and *g* is called to be satisfy (H1) if for every (*z*, *x*)∈*W*, there exist a neighborhood *V* ⊆ *W* of (*z*, *x*) and constants *L*_0_, such that

$$\begin{array}{c} \|f(s,w)-f(s,v)\|\leq L_{0}{\left\|w-v\right\|}_{\alpha },\;\;\;\text{for}\;\text{some}\;\;\left(s,w\right)\;\;\;\text{and}\;\;\left(s,v\right)\;\;\text{on}\;\;V. \end{array}$$

$$\begin{array}{c} \|g(s,w)-g(s,v)\|\leq L_{0}{\left\|w-v\right\|}_{\alpha },\;\;\;\text{for}\;\text{some}\;\;\left(s,w\right)\;\;\;\text{and}\;\;\left(s,v\right)\;\;\text{on}\;\;V. \end{array}$$

(H2) It is possible to assume without losing generality that IGAS by −*ζ* is bounded and that −*ζ* is invertible. In addition, suppose that 0 < *T* < ∞ to establish local existence.

(H3) *I*_*k*_ ∈ *C*(*X*_*α*_, *X*_*α*_), *k* = 1, 2, …, *m*, and Ψ_*k*_ ∈ *C*(*I*, *R*_+_), *k* = 1, 2, …, *m* is nondecreasing maps and

$$\begin{array}{c} {\left\|I_{k}\left(x\right)\right\|}_{\alpha }\leq \Psi_{k}\left({\left\|x\right\|}_{\alpha }\right),\;\text{for}\;x\in X_{\alpha } \end{array}$$

There exists *ρ* > 0 and

$$\begin{array}{c} \|I_{k}\left(x\right)-I_{k}\left(y\right)\|\leq \rho {\left\|x-y\right\|}_{Y},\;\text{for}\;x,y\in X_{\alpha } \end{array}$$

(H3′) there exists *ρ*_1_ > 0 and

$$\begin{array}{c} \|\varphi (x)-\varphi (y)\|\leq \rho_{1}{\left\|x-y\right\|}_{Y},\;\text{for}\;x,y\in X_{\alpha }. \end{array}$$

The notations will be continued considered in the previous section.

An equivalent integral equation for *z* ≥ *z*_0_ is associated with the existence of a solution to (1) as the following:

$$\begin{array}{c} w(z)=G\left(z-z_{0}\right)\left(w_{0}-\varphi \left(w\right)\right)+\sum_{z_{0}<z_{k}<z}G\left(z-z_{k}\right)I_{k}\left(w\left(z_{k}^{-}\right)\right)\\ +\frac{1}{\Gamma (\eta )}\int_{z_{0}}^{z}{\left(z-s\right)}^{\eta -1}G\left(z-s\right)(f\left(s,w\left(s\right)\right)+\int_{z_{0}}^{s}q\left(s-\tau \right)g\left(\tau ,w\left(\tau \right)\right)d\tau )\;ds. \end{array}$$

The following theorem follows from these simplifications.

**Theorem 3.1** *Let* −*ζ* *be the operator generates the analytic-semigroup* *G*(*z*) *where* 0 ∈ *ρ*(−*ζ*). *If the maps* *f* *and* *g* *satisfy (H1) and* *q* *be the real valued map where it integrable in* *I*, *then the* Eq (1) *has a unique local mild solution for every* *w*_0_ ∈ *X*_*α*_. *Therefore, if the kernel* *q* *satisfied the condition blow*

*(H4) There exist positive constants* *C*_0_ > 0 *and* 0 < *β* ≤ 1 *such that*

$$\begin{array}{c} \left|q\left(z\right)-q\left(s\right)\right|\leq C_{0}{\left|z-s\right|}^{\beta }, \end{array}$$

*for all* *z*, *s* ∈ *I*. *Hence the mild solutionis also Hölder continuous*.

**Proof.** We will use the concepts and notations introduced in the previous section. Let (*z*_0_, *w*_0_) be a point in an open subset *U* of [0, ∞) × *X*_*α*_ and choose $z_{1}^{\prime }>z_{0}$ such that (H1) satisfied, with some positive number *L*_0_ > 0 holds for the set of maps *f* and *g*

$$\begin{array}{c} V=\{\left(z,x\right)\in U:z_{0}\leq z\leq z_{1}^{\prime },{\left\|x-(w_{0}-\varphi \left(y\right))\right\|}_{\alpha }\leq r\}. \end{array}$$

Let

$$\begin{array}{c} B_{1}=\underset{z_{0}\leq z\leq z_{1}^{\prime }}{\text{sup}}\|f(z,w_{0}-\varphi \left(y\right))\| \end{array}$$

and

$$\begin{array}{c} B_{2}=\underset{t_{0}\leq z\leq z_{1}^{\prime }}{\text{sup}}\|g(z,w_{0}-\varphi \left(y\right))\|. \end{array}$$

Choose *τ* > *tz*_0_ such that

$$\begin{array}{c} \|G(z-z_{0})-i\|\|\zeta^{\alpha }\left(w_{0}-\varphi \left(y\right)\right)\|+\;\sum_{k=1}^{m}\frac{C_{\alpha }\Psi_{k}\left(r\right)}{{\left(\tau -z_{k}\right)}^{\alpha }}\leq \frac{r}{2},\;\text{for}\;z_{0}\leq z\leq \tau \end{array}$$

$$\begin{array}{c} \frac{C_{\alpha }\rho_{1}}{{\left(z-z_{0}\right)}^{\alpha }}+\;\sum_{k=1}^{m}\frac{C_{\alpha }\rho }{{\left(z-z_{k}\right)}^{\alpha }}\leq \frac{1}{4} \end{array}$$

and

$$\begin{array}{c} \tau -z_{0}<\text{min}\{z_{1}^{\prime }-z_{0},{\left[\frac{r}{2}\;C_{\alpha }^{-1}\left(\eta -\alpha \right)\Gamma \left(\eta \right){\left[L\left(r\right)\right]}^{-1}\right]}^{\frac{1}{\eta -\alpha }}\}, \end{array}$$ (2)

where *L*(*r*) = (*L*_0_*r* + *B*_1_) + *a*_*T*_(*L*_0_*r* + *B*_2_) and *C*_*α*_ is a positive constant depending on *α* satisfying

$$\begin{array}{c} \|\zeta^{\alpha }G\left(z\right)\|\leq C_{\alpha }z^{-\alpha },\;\text{for}\;z>z_{0}\;\text{and}\;a_{T}=\int_{z_{0}}^{T}\left|q\left(s\right)\right|ds. \end{array}$$ (3)

Let endow *Y* = *C*([*z*_0_, *τ*];*X*) with the supremum norm

$$\begin{array}{c} {\left\|y\right\|}_{Y}=\underset{z_{0}\leq z\leq \tau }{\text{sup}}\|y(z)\|. \end{array}$$

Thus *Y* is a BS. Then *Fy* be a map on *Y* and is given by

$$\begin{array}{cc} & Fy\left(z\right)=G\left(z-z_{0}\right)\zeta^{\alpha }\left(w_{0}-\varphi \left(y\right)\right)+\sum_{z_{0}<z_{k}<z}\zeta^{\alpha }G\left(z-z_{k}\right)I_{k}\left(y\left(z_{k}^{-}\right)\right)\\ & +\frac{1}{\Gamma (\eta )}\int_{z_{0}}^{z}{\left(z-s\right)}^{\eta -1}\zeta^{\alpha }G\left(z-s\right)(f\left(s,\zeta^{-\alpha }y\left(s\right)\right)+\int_{z_{0}}^{s}q\left(s-\tau \right)g\left(\tau ,\zeta^{-\alpha }y\left(\tau \right)\right)d\tau )\;ds. \end{array}$$

It clear by hypothesis (H1) on the functions *f* and *g*, and condition (3) that *F*: *Y* → *Y* and ∀*y* ∈ *Y*, *Fy*(*z*_0_) = *ζ*^*α*^(*w*_0_−*φ*(*y*)).

Let Ω be the non-empty bounded and closed set given by

$$\begin{array}{c} \Omega =\{y\in Y:y\left(z_{0}\right)=\zeta^{\alpha }\left(w_{0}-\varphi \left(y\right)\right),\;\|y\left(z\right)-\zeta^{\alpha }\left(w_{0}-\varphi \left(y\right)\right)\|\leq r\}. \end{array}$$

Then for *y* ∈ Ω, we have

$$\begin{array}{c} \left\|Fy\left(z\right)-\zeta^{\alpha }\left(w_{0}-\varphi \left(y\right)\right)\|\leq \|G(z-z_{0})-i\|\|\zeta^{\alpha }\left(w_{0}-\varphi \left(y\right)\right)\right\|\\ +\frac{1}{\Gamma (\eta )}\int_{z_{0}}^{z}{\left(z-s\right)}^{\eta -1}\|\zeta^{\alpha }G\left(z-s\right)\|(\|f\left(s,\zeta^{-\alpha }y\left(s\right)\right)-f\left(s,w_{0}-\varphi \left(y\right)\right)\|)\;ds\\ +\frac{1}{\Gamma (\eta )}\int_{z_{0}}^{z}{\left(z-s\right)}^{\eta -1}\|\zeta^{\alpha }G\left(z-s\right)\|(\|f(s,w_{0}-\varphi \left(y\right))\|)\;ds\\ +\frac{1}{\Gamma (\eta )}\int_{z_{0}}^{z}{\left(z-s\right)}^{\eta -1}\|\zeta^{\alpha }G\left(z-s\right)\|(\int_{z_{0}}^{s}\left|q\left(s-\tau \right)\right|\|g\left(\tau ,\zeta^{-\alpha }y\left(\tau \right)\right)-g\left(\tau ,w_{0}-\varphi \left(y\right)\right)\|d\tau )\;ds\\ +\frac{1}{\Gamma (\eta )}\int_{t_{0}}^{z}{\left(z-s\right)}^{\eta -1}\|\zeta^{\alpha }G\left(z-s\right)\|(\int_{z_{0}}^{s}\left|q\left(s-\tau \right)\right|\|g(\tau ,g\left(\tau ,w_{0}-\varphi \left(y\right)\right)\|d\tau )\;ds\\ +\sum_{z_{0}<z_{k}<z}\|\zeta^{\alpha }G\left(z-z_{k}\right)\|\|I_{k}\left(y\left(z_{k}^{-}\right)\right)\|\\ \leq \|G(z-z_{0})-i\|\|\zeta^{\alpha }\left(w_{0}-\varphi \left(y\right)\right)\|+\;\sum_{k=1}^{m}\frac{C_{\alpha }\Psi_{k}\left(r\right)}{{\left(\tau -z_{k}\right)}^{\alpha }}\\ +\frac{C_{\alpha }{\left(\tau -z_{0}\right)}^{\eta -\alpha }}{\left(\eta -\alpha )\Gamma (\eta \right)}\left[\left(L_{0}r+B_{1}+a_{T}\left(L_{0}r+B_{2}\right)\right)\right]\\ \leq \frac{r}{2}+\frac{C_{\alpha }{\left(\tau -z_{0}\right)}^{\eta -\alpha }}{\left(\eta -\alpha )\Gamma (\eta \right)}[L(r)]\\ \leq r. \end{array}$$

where the last two inequalities follows from above assumption. Hence, we have *F*: Ω → Ω. We can see that *F* is a strict contraction in Ω. There will be a unique continuous function satisfying this requirement (1). For *y* ∈ Ω and *θ* ∈ Ω, then

$$\begin{array}{c} \|Fy(z)-F\theta (z)\|\leq \|G\left(z-z_{0}\right)\zeta^{\alpha }\|\|(\varphi (\theta )-\varphi (y))\|\\ +\frac{1}{\Gamma (\eta )}\int_{z_{0}}^{z}{\left(z-s\right)}^{\eta -1}\|\zeta^{\alpha }G\left(z-s\right)\|\|f\left(s,\zeta^{-\alpha }y\left(s\right)\right)-f\left(s,\zeta^{-\alpha }\theta \left(s\right)\right)\|\;ds\\ +\frac{1}{\Gamma (\eta )}\int_{z_{0}}^{z}{\left(z-s\right)}^{\eta -1}\|\zeta^{\alpha }G\left(z-s\right)\|\\ \times (\int_{z_{0}}^{s}\left|q\left(s-\tau \right)\right|\|g\left(\tau ,\zeta^{-\alpha }y\left(\tau \right)\right)-g\left(\tau ,\zeta^{-\alpha }\theta \left(\tau \right)\right)\|d\tau )\;ds\\ +\sum_{z_{0}<z_{k}<z}\|\zeta^{\alpha }G\left(z-z_{k}\right)\|\|I_{k}\left(y\left(z_{k}^{-}\right)\right)-I_{k}\left(\theta \left(z_{k}^{-}\right)\right)\|. \end{array}$$

Assuming (H1) on *g* and *f* and condition (3) we get

$$\begin{array}{c} \|Fy(z)-F\theta (z)\|\leq \frac{C_{\alpha }\rho_{1}}{{\left(z-z_{0}\right)}^{\alpha }}{\left\|y-\theta \right\|}_{Y}\\ +\frac{L_{0}\left(1+a_{T}\right)}{\Gamma (\eta )}\int_{z_{0}}^{z}{\left(z-s\right)}^{\eta -1}\|\zeta^{\alpha }G\left(z-s\right)\|\;ds{\left\|y-\theta \right\|}_{Y}\\ +\sum_{z_{0}<z_{k}<z}\frac{C_{\alpha }\rho }{{\left(z-z_{k}\right)}^{\alpha }}{\left\|y-\theta \right\|}_{Y}\\ \leq (\frac{C_{\alpha }\rho_{1}}{{\left(z-z_{0}\right)}^{\alpha }}+\frac{L_{0}\left(1+a_{T}\right)C_{\alpha }}{\left(\eta -\alpha )\Gamma (\eta \right)}{\left(z-z_{0}\right)}^{\eta -\alpha }+\;\sum_{k=1}^{m}\frac{C_{\alpha }\rho }{{\left(z-z_{k}\right)}^{\alpha }}){\left\|y-\theta \right\|}_{Y}\\ \leq (\frac{rL_{0}\left(1+a_{T}\right)}{2L(r)}+\frac{C_{\alpha }\rho_{1}}{{\left(z-z_{0}\right)}^{\alpha }}+\;\sum_{k=1}^{m}\frac{C_{\alpha }\rho }{{\left(z-z_{k}\right)}^{\alpha }}){\left\|y-\theta \right\|}_{Y}\\ \leq (\;\frac{1}{2}+\frac{1}{4}){\left\|y-\theta \right\|}_{Y}\\ \leq \frac{3}{4}{\left\|y-\theta \right\|}_{Y}. \end{array}$$

In the last inequality we use (2). Then the map *F* is a strict contraction where *F*: Ω → Ω and hence we have a unique fixed point *y* of *F* in Ω, i.e. there is a unique *y* ∈ Ω such that *Fy* = *y*, by using the Banach contraction principle.

Suppose that *w* = *ζ*^−*α*^*y*. Thus for *z* ∈ [*z*_0_, *τ*], we have

$$\begin{array}{c} w(z)=\zeta^{-\alpha }y\left(z\right)\\ =G\left(z-z_{0}\right)\left(w_{0}-\varphi \left(w\right)\right)+\sum_{z_{0}<z_{k}<z}G\left(z-z_{k}\right)I_{k}\left(w\left(z_{k}^{-}\right)\right)\\ +\frac{1}{\Gamma (\eta )}\int_{z_{0}}^{z}{\left(z-s\right)}^{\eta -1}G\left(z-s\right)(f(s,w(s))+K(w)(s))\;ds. \end{array}$$

This follow that *w* is a unique local mild solution to (1).

Thus for the kernel *q* satisfies the condition (H4), we show the Hölder continuity of the solution. Since there exist *T*_0_, *z*_0_ < *T*_0_ < *T* and a map *w* where *w* is a mild unique solution to (1) on *I*_0_ = (*z*_0_, *T*_0_) defined by

$$\begin{array}{c} w(z)=G\left(z-z_{0}\right)\left(w_{0}-\varphi \left(w\right)\right)+\sum_{z_{0}<z_{k}<z}G\left(z-z_{k}\right)I_{k}\left(w\left(z_{k}^{-}\right)\right),\;z\in I_{0}\\ +\frac{1}{\Gamma (\eta )}\int_{z_{0}}^{z}{\left(z-s\right)}^{\eta -1}G\left(z-s\right)(f(s,w(s))+K(w)(s))\;ds, \end{array}$$

where

$$\begin{array}{c} K\left(w\right)\left(z\right)=\int_{z_{0}}^{z}q\left(z-s\right)g\left(s,w\left(s\right)\right)ds. \end{array}$$

For *z*_0_ < *τ*_1_ < *τ*_2_ < *T* and let *v*(*z*) = *ζ*^*α*^*w*(*z*). Then,

$$\begin{array}{cc} & v\left(z\right)=G\left(z-z_{0}\right)\zeta^{\alpha }\left(w_{0}-\varphi \left(w\right)\right)+\sum_{z_{0}<z_{k}<z}\zeta^{\alpha }G\left(z-z_{k}\right)I_{k}\left(v\left(z_{k}^{-}\right)\right)\\ & +\frac{1}{\Gamma (\eta )}\int_{z_{0}}^{z}{\left(z-s\right)}^{\eta -1}\zeta^{\alpha }G\left(z-s\right)(f\left(s,\zeta^{-\alpha }v\left(s\right)\right)+\int_{z_{0}}^{s}q\left(s-\tau \right)g\left(\tau ,\zeta^{-\alpha }v\left(\tau \right)\right)d\tau )\;ds. \end{array}$$

For simplification, set

$$\begin{array}{c} \tilde{f}\left(z\right)=f\left(z,\zeta^{-\alpha }v\left(z\right)\right),\;\;\tilde{g}\left(z\right)=g\left(z,\zeta^{-\alpha }v\left(z\right)\right). \end{array}$$

Then the above equation can be rewritten as

$$\begin{array}{c} v(z)=G\left(z-z_{0}\right)\zeta^{\alpha }\left(w_{0}-\varphi \left(w\right)\right)+\sum_{z_{0}<z_{k}<z}\zeta^{\alpha }G\left(z-z_{k}\right)I_{k}\left(v\left(z_{k}^{-}\right)\right)\\ +\frac{1}{\Gamma (\eta )}\int_{z_{0}}^{z}{\left(z-s\right)}^{\eta -1}\zeta^{\alpha }G\left(z-s\right)(\tilde{f}\left(s\right)+\int_{z_{0}}^{s}q\left(s-\tau \right)\tilde{g}\left(\tau \right)d\tau )\;ds. \end{array}$$

Since the maps *f* and *g* satisfy assumption (H1) and *w*(*z*) is continuous on *I*_0_, it clear that $\tilde{f}$ and $\tilde{g}$ are continuous. Hence bounded on *I*_0_ and let

$$\begin{array}{c} N_{1}=\underset{z\in J_{0}}{\text{sup}}\|\tilde{f}\left(z\right)\|\;\text{and}\;N_{2}=\underset{t\in I_{0}}{\text{sup}}\|\tilde{g}\left(t\right)\|\;\text{and}\;S=\underset{w\in X_{\alpha }}{\text{sup}}\|\varphi (w)\| \end{array}$$

$$\begin{array}{c} v(\tau_{2})-v\left(\tau_{1}\right)=\left(G\left(\tau_{2}-z_{0}\right)-G\left(\tau_{1}-z_{0}\right)\right)\zeta^{\alpha }\left(w_{0}-\varphi \left(w\right)\right)\\ +\frac{1}{\Gamma (\eta )}\int_{z_{0}}^{\tau_{1}}{\left(\tau_{1}-s\right)}^{\eta -1}\zeta^{\alpha }G\left(\tau_{2}-s\right)(\tilde{f}\left(s\right)+\int_{z_{0}}^{s}q\left(s-\tau \right)\tilde{g}\left(\tau \right)d\tau )\;ds\\ +\frac{1}{\Gamma (\eta )}\int_{z_{0}}^{\tau_{2}}{\left(\tau_{2}-s\right)}^{\eta -1}\zeta^{\alpha }G\left(\tau_{2}-s\right)(\tilde{f}\left(s\right)+\int_{z_{0}}^{s}q\left(s-\tau \right)\tilde{g}\left(\tau \right)d\tau )\;ds\\ -\frac{1}{\Gamma (\eta )}\int_{z_{0}}^{\tau_{1}}{\left(\tau_{1}-s\right)}^{\eta -1}\zeta^{\alpha }G\left(\tau_{1}-s\right)(\tilde{f}\left(s\right)+\int_{z_{0}}^{s}q\left(s-\tau \right)\tilde{g}\left(\tau \right)d\tau )\;ds\\ +\sum_{tz_{0}<t_{k}<\tau_{2}}\zeta^{\alpha }G\left(\tau_{2}-z_{k}\right)I_{k}\left(v\left(z_{k}^{-}\right)\right)-\sum_{z_{0}<z_{k}<\tau_{1}}\zeta^{\alpha }G\left(\tau_{1}-z_{k}\right)I_{k}\left(v\left(z_{k}^{-}\right)\right)\\ =\varepsilon_{1}+\varepsilon_{2}+\varepsilon_{3}+\varepsilon_{4}+\varepsilon_{5}. \end{array}$$

Where,

$$\begin{array}{c} \varepsilon_{1}=\left(G\left(\tau_{2}-z_{0}\right)-G\left(\tau_{1}-z_{0}\right)\right)\zeta^{\alpha }\left(w_{0}-\varphi \left(w\right)\right)\\ \varepsilon_{2}=\frac{1}{\Gamma (\eta )}\int_{\tau_{1}}^{\tau_{2}}{\left(\tau_{2}-s\right)}^{\eta -1}\zeta^{\alpha }G\left(\tau_{2}-s\right)(\tilde{f}\left(s\right)+\int_{z_{0}}^{s}q\left(s-\tau \right)\tilde{g}\left(\tau \right)d\tau )\;ds\\ \varepsilon_{3}=\frac{1}{\Gamma (\eta )}\int_{z_{0}}^{\tau_{1}}\left[{\left(\tau_{1}-s\right)}^{\eta -1}-{\left(\tau_{2}-s\right)}^{\eta -1}\right]\zeta^{\alpha }G\left(\tau_{2}-s\right)(\tilde{f}\left(s\right)+\int_{z_{0}}^{s}q\left(s-\tau \right)\tilde{g}\left(\tau \right)d\tau )\;ds\\ \varepsilon_{4}=\frac{1}{\Gamma (\eta )}\int_{z_{0}}^{\tau_{1}}{\left(\tau_{1}-s\right)}^{\eta -1}\zeta^{\alpha }\left[G\left(\tau_{2}-s\right)-G\left(\tau_{1}-s\right)\right](\tilde{f}\left(s\right)+\int_{z_{0}}^{s}q\left(s-\tau \right)\tilde{g}\left(\tau \right)d\tau )\;ds\\ \varepsilon_{5}=\sum_{\tau_{1}<z_{k}<\tau_{2}}\zeta^{\alpha }G\left(\tau_{2}-z_{k}\right)I_{k}\left(v\left(z_{k}^{-}\right)\right)+\sum_{z_{0}<z_{k}<\tau_{1}}\zeta^{\alpha }\left[G\left(\tau_{2}-z_{k}\right)-G\left(\tau_{1}-z_{k}\right)\right]I_{k}\left(v\left(z_{k}^{-}\right)\right), \end{array}$$

and

$$\begin{array}{c} \|v\left(\tau_{2}\right)-v\left(\tau_{1}\right)\|\leq \|\varepsilon_{1}\|+\|\varepsilon_{2}\|+\|\varepsilon_{3}\|+\|\varepsilon_{4}\|+\|\varepsilon_{5}\|, \end{array}$$

we have,

$$\begin{array}{cc} \|\varepsilon_{1}\|\leq & \left\|\left(G\left(\tau_{2}-z_{0}\right)-G\left(\tau_{1}-z_{0}\right)\right)\zeta^{\alpha }\left(w_{0}-\varphi \left(w\right)\right)\right\|\\ \leq & C_{\beta }C_{\alpha +\beta }{\left(\tau_{2}-\tau_{1}\right)}^{\beta }{\left(\tau_{1}-z_{0}\right)}^{-(\alpha +\beta )}\|w_{0}-\varphi \left(w\right))\|\\ \leq & C_{\beta }C_{\alpha +\beta }{\left(\tau_{2}-\tau_{1}\right)}^{\beta }{\left(\tau_{1}-z_{0}\right)}^{-(\alpha +\beta )}\left(\|w_{0}\|+S\right), \end{array}$$

from which we deduce $\underset{\tau_{1}\to \tau_{2}}{\text{lim}}\varepsilon_{1}=0.$

$$\begin{array}{c} \|\varepsilon_{2}\|=\frac{1}{\Gamma (\eta )}\int_{\tau_{1}}^{\tau_{2}}{\left(\tau_{2}-s\right)}^{\eta -1}\|\zeta^{\alpha }G\left(\tau_{2}-s\right)\|(\tilde{f}\left(s\right)+\int_{z_{0}}^{s}q\left(s-\tau \right)\tilde{g}\left(\tau \right)d\tau )\;ds\\ \leq \frac{C_{\alpha }}{\Gamma (\eta )}\int_{\tau_{1}}^{\tau_{2}}{\left(\tau_{2}-s\right)}^{\eta -\alpha -1}(N_{1}+a_{T_{0}}N_{2}T_{0})\;ds\\ \|\varepsilon_{2}\|\leq \frac{C_{\alpha }\left[N_{1}+a_{T_{0}}N_{2}T_{0}\right]}{\left(\eta -\alpha )\Gamma (\eta \right)}{\left(\tau_{2}-\tau_{1}\right)}^{\eta -\alpha }. \end{array}$$

Hence $\underset{\tau_{1}\to \tau_{2}}{\text{lim}}\varepsilon_{2}=0.$

$$\begin{array}{c} \|\varepsilon_{3}\|=\frac{1}{\Gamma (\eta )}\int_{z_{0}}^{\tau_{1}}\left[{\left(\tau_{1}-s\right)}^{\eta -1}-{\left(\tau_{2}-s\right)}^{\eta -1}\right]\|\zeta^{\alpha }G\left(\tau_{2}-s\right)\|(\tilde{f}\left(s\right)+\int_{z_{0}}^{s}q\left(s-\tau \right)\tilde{g}\left(\tau \right)d\tau )\;ds\\ \|\varepsilon_{3}\|\leq \frac{\left[N_{1}+a_{T_{0}}N_{2}T_{0}\right]}{\Gamma (\eta )}\int_{z_{0}}^{\tau_{1}}\left[{\left(\tau_{1}-s\right)}^{\eta -1}-{\left(\tau_{2}-s\right)}^{\eta -1}\right]\|\zeta^{\alpha }G\left(\tau_{2}-s\right)\|\;ds\\ \leq \frac{\left[N_{1}+a_{T_{0}}N_{2}T_{0}\right]}{\Gamma (\eta )}\int_{z_{0}}^{\tau_{1}}\left[{\left(\tau_{1}-s\right)}^{\eta -1}-{\left(\tau_{2}-s\right)}^{\eta -1}\right]C_{\alpha }{\left(\tau_{2}-s\right)}^{-\alpha }\;ds\\ \leq \frac{C_{\alpha }T^{-\alpha }\left[N_{1}+a_{T_{0}}N_{2}T_{0}\right]}{\Gamma (\eta )}\int_{z_{0}}^{\tau_{1}}[{\left(\tau_{1}-s\right)}^{\eta -1}-{\left(\tau_{2}-s\right)}^{\eta -1}\;ds]\\ \leq \frac{C_{\alpha }T^{-\alpha }\left[N_{1}+a_{T_{0}}N_{2}T_{0}\right]}{\eta \Gamma (\eta )}{\left|\tau_{2}-\tau_{1}\right|}^{\eta }. \end{array}$$

Hence $\underset{\tau_{1}\to \tau_{2}}{\text{lim}}\varepsilon_{3}=0.$

$$\begin{array}{c} \|\varepsilon_{4}\|=\frac{1}{\Gamma (\eta )}\int_{z_{0}}^{\tau_{1}}\|{\left(\tau_{1}-s\right)}^{\eta -1}\zeta^{\alpha }\left[G\left(\tau_{2}-s\right)-G\left(\tau_{1}-s\right)\right]\|(\tilde{f}\left(s\right)+\int_{z_{0}}^{s}q\left(s-\tau \right)\tilde{g}\left(\tau \right)d\tau )\;ds\\ \leq \frac{\left[N_{1}+a_{T_{0}}N_{2}T_{0}\right]}{\Gamma (\eta )}\int_{z_{0}}^{\tau_{1}}\|{\left(\tau_{1}-s\right)}^{\eta -1}\zeta^{\alpha }\left[G\left(\tau_{2}-s\right)-G\left(\tau_{1}-s\right)\right]\|\;ds\\ \leq \frac{T^{\eta -1}\left[N_{1}+a_{T_{0}}N_{2}T_{0}\right]}{\Gamma (\eta )}\int_{z_{0}}^{\tau_{1}}\|\zeta^{\alpha }\left[G\left(\tau_{2}-s\right)-G\left(\tau_{1}-s\right)\right]\|\;ds\\ \leq \frac{T^{\eta -1}\left[N_{1}+a_{T_{0}}N_{2}T_{0}\right]}{\Gamma (\eta )}\int_{t_{0}}^{\tau_{1}}\|[G(\frac{\tau_{2}-\tau_{1}}{2}+\frac{\tau_{2}-s}{2})-G(\frac{\tau_{1}-s}{2})]\zeta^{\alpha }G(\frac{\tau_{1}-s}{2})\|\;ds\\ \leq \frac{C_{\alpha }T^{\eta -\alpha -1}\left[N_{1}+a_{T_{0}}N_{2}T_{0}\right]}{\Gamma (\eta )}\int_{z_{0}}^{\tau_{1}}\|[G(\frac{\tau_{2}-\tau_{1}}{2}+\frac{\tau_{2}-s}{2})-G(\frac{\tau_{1}-s}{2})]\|\;ds. \end{array}$$

Therefore, the continuity of the function *z* → ‖*G*(*z*)‖ for *z* ∈ (0, *T*) allows us to conclude that $\underset{\tau_{1}\to \tau_{2}}{\text{lim}}\varepsilon_{4}=0.$ For *k* = 1, …, *m*, we can write

$$\begin{array}{c} \|\varepsilon_{5}\|=\\ \left\|\sum_{\tau_{1}<z_{k}<\tau_{2}}\zeta^{\alpha }G\left(\tau_{2}-z_{k}\right)I_{k}\left(v\left(z_{k}^{-}\right)\right)+\sum_{z_{0}<z_{k}<\tau_{1}}\zeta^{\alpha }\left[G\left(\tau_{2}-z_{k}\right)-G\left(\tau_{1}-z_{k}\right)\right]I_{k}\left(v\left(z_{k}^{-}\right)\right)\right\|\\ \leq \left\|\sum_{\tau_{1}<z_{k}<\tau_{2}}\zeta^{\alpha }G\left(\tau_{2}-z_{k}\right)I_{k}\left(v\left(z_{k}^{-}\right)\right)\|+\|\sum_{z_{0}<z_{k}<\tau_{1}}\zeta^{\alpha }\left[G\left(\tau_{2}-z_{k}\right)-G\left(\tau_{1}-z_{k}\right)\right]I_{k}\left(v\left(z_{k}^{-}\right)\right)\right\|\\ \leq \|\sum_{\tau_{1}<z_{k}<\tau_{2}}\zeta^{\alpha }G\left(\tau_{2}-z_{k}\right)I_{k}\left(v\left(z_{k}^{-}\right)\right)\|+\\ \|\sum_{z_{0}<z_{k}<\tau_{1}}[G(\frac{\tau_{2}-z_{k}}{2}+\frac{\tau_{2}-\tau_{1}}{2})-G(\frac{\tau_{1}-z_{k}}{2})]\;\zeta^{\alpha }G(\frac{\tau_{1}-z_{k}}{2})I_{k}\left(v\left(z_{k}^{-}\right)\right)\|. \end{array}$$

Hence $\underset{\tau_{1}\to \tau_{2}}{\text{lim}}\varepsilon_{5}=0.$ Thus from the above inequality Hölder the continuity of *v*(*z*) follows.

## 4 Global existence

We need the following lemma to find the global existence of a mild solution to (1).

**Lemma 4.1** [23] *Let* *φ*(*z*, *s*) = *w*_0_(*z*, *s*) + *φ*(*w*(*z*, *s*)) ≥ 0 *be continuous function on* 0 ≤ *s* ≤ *z* ≤ *T* < ∞. *If there exist two positive constant* *L*, *M* *and* *α* *where*

$$\begin{array}{c} \varphi \left(z,s\right)\leq L+M\int_{s}^{z}{\left(z-\sigma \right)}^{\alpha -1}\;\;\varphi \left(\sigma ,s\right)\;d\sigma \end{array}$$

*then there is a constant* *N* *and* *φ*(*z*, *s*)≤*N*, *for* 0 ≤ *s* ≤ *z* ≤ *T*.

The following theorem prove that there is a global existence of a mild solution to (1).

**Theorem 4.1** *Let* 0 ∈ *D*(*ζ*) *and let* −*ζ* *be IGAS* *G*(*t*) *satisfying* ‖*G*(*z*)‖ ≤ *M* *for* *z* ≥ *z*_0_. Let *f*, *g*: [*z*_0_, ∞) × *X*_*α*_ → *X* *satisfy assumption* *H*1. *If there is continuous discreasing function* *k*_1_ *and* *k*_2_ *from* [*z*_0_, ∞) *into* [0, ∞) *such that*

$$\begin{array}{c} \|f(z,s)\|\leq K_{1}\left(1+{\left\|x\right\|}_{\alpha }\right),\;\text{for}\;z\geq z_{0},x\in X_{\alpha }, \end{array}$$

$$\begin{array}{c} \|g(z,s)\|\leq K_{2}\left(1+{\left\|x\right\|}_{\alpha }\right),\;\text{for}\;z\geq z_{0},x\in X_{\alpha }, \end{array}$$

*then the impulsive nonlocal problem* (1) *has a unique mild solution* *w* *on* [*z*_0_, ∞) *for every* *w*_0_−*φ*(*w*)∈*X*_*α*_.

**Proof.** By Theorem 3.1 we have a *T*_0_, *z*_0_ < *T*_0_ and *w* on *I*_0_ = [*z*_0_, *T*_0_] be a unique solution. For some positive constant *C*, if ‖*w*(*z*)‖_*α*_ ≤ *C* for *z* ∈ *I*_0_, then on the right of *T*_0_ the solution *w*(*z*) may be continued on it. Therefor, it suffices to show that if on [*z*_0_, *T*], *z*_0_ < *T* < ∞ there is a mild solution *w* to (1), then ‖*w*(*z*)‖_*α*_ is bounded as *z* ↑ *T*. Since *w*(*z*) is a mild solution. Hence we have

$$\begin{array}{c} w(z)=G\left(z-z_{0}\right)\left(w_{0}-\phi \left(w\right)\right)+\sum_{z_{0}<z_{k}<z}G\left(z-z_{k}\right)I_{k}\left(w\left(z_{k}^{-}\right)\right)\\ +\frac{1}{\Gamma (\eta )}\int_{z_{0}}^{z}{\left(z-s\right)}^{\eta -1}G\left(z-s\right)(f\left(s,w\left(s\right)\right)+\int_{z_{0}}^{s}q\left(s-\tau \right)g\left(\tau ,w\left(\tau \right)\right)d\tau )\;ds. \end{array}$$

By the fact that *G*(*z*) commutes with *ζ* and that ‖*G*(*z*)‖ ≤ *M*, ‖*ζ*^*α*^*G*(*z*)‖ ≤ *C*_*α*_*z*^−*α*^ for *z* ≥ *z*_0_ in above equation taking norms of both sides and applying *ζ*^*α*^, we have

$$\begin{array}{c} {\left\|w(z)\right\|}_{\alpha }=\left\|\zeta^{\alpha }G\left(z-z_{0}\right)\left(w_{0}-\varphi \left(w\right)\right)\|+\sum_{z_{0}<z_{k}<z}\|\zeta^{\alpha }G\left(z-z_{k}\right)I_{k}\left(w\left(z_{k}^{-}\right)\right)\right\|\\ +\frac{1}{\Gamma (\eta )}\int_{z_{0}}^{z}\|{\left(z-s\right)}^{\eta -1}\zeta^{\alpha }G\left(z-s\right)(f\left(s,w\left(s\right)\right)+\int_{z_{0}}^{s}q\left(s-\tau \right)g\left(\tau ,w\left(\tau \right)\right)d\tau )\;ds\|\\ \leq M\|\zeta^{\alpha }\left(w_{0}-\varphi \left(y\right)\right)\|+\;\sum_{k=1}^{m}\frac{C_{\alpha }\Psi_{k}\left(r\right)}{{\left(z-z_{k}\right)}^{\alpha }}\\ +\frac{C_{\alpha }}{\Gamma (\eta )}\int_{z_{0}}^{z}\|{\left(z-s\right)}^{\eta -\alpha -1}f\left(s,w\left(s\right)\right)+\int_{z_{0}}^{s}q\left(s-\tau \right)g\left(\tau ,w\left(\tau \right)\right)d\tau \|\;ds. \end{array}$$

For the last equation we have the estimate

$$\begin{array}{c} \int_{z_{0}}^{s}\left|q\left(s-\tau \right)\right|\|g(\tau ,w(\tau ))d\tau \|\leq a_{T}k_{2}\left(T\right)\int_{z_{0}}^{s}\left(1+{\left\|w(\tau )\right\|}_{\alpha }\right)\;d\tau . \end{array}$$

For the equation incorporating the estimate, we have

$$\begin{array}{cc} & {\left\|w(z)\right\|}_{\alpha }\leq M\|\zeta^{\alpha }\left(w_{0}-\varphi \left(y\right)\right)\|+\;\sum_{k=1}^{m}\frac{C_{\alpha }\Psi_{k}\left(r\right)}{{\left(z-z_{k}\right)}^{\alpha }}\\ & +\frac{C_{\alpha }\left[K_{1}\left(T\right)+a_{T}K_{2}\left(z\right)\right]}{\Gamma (\eta )}\int_{z_{0}}^{z}{\left(z-s\right)}^{\eta -\alpha -1}(1+{\left\|w(s)\right\|}_{\alpha }+\int_{z_{0}}^{s}\left(1+{\left\|w(\tau )\right\|}_{\alpha }\right)\;d\tau )\;ds. \end{array}$$

After a slight modification in the equation, we have

$$\begin{array}{cc} & {\left\|w(z)\right\|}_{\alpha }\leq M\|\zeta^{\alpha }\left(w_{0}-\varphi \left(y\right)\right)\|+\;\sum_{k=1}^{m}\frac{C_{\alpha }\Psi_{k}\left(r\right)}{{\left(z-z_{k}\right)}^{\alpha }}\\ & +\frac{C_{\alpha }\left(1+T\right)\left[K_{1}\left(T\right)+a_{T}K_{2}\left(z\right)\right]T^{\eta -\alpha }}{\Gamma (\eta )(\eta -\alpha )}\int_{z_{0}}^{z}{\left(z-s\right)}^{\eta -\alpha -1}({\left\|w(s)\right\|}_{\alpha }+\int_{z_{0}}^{s}{\left\|w(\tau )\right\|}_{\alpha }\;d\tau )\;ds. \end{array}$$

The estimate in the equation is

$$\begin{array}{c} {\left\|w(z)\right\|}_{\alpha }\leq C_{1}+C_{2}\int_{z_{0}}^{z}{\left(z-s\right)}^{\eta -\alpha }({\left\|w(s)\right\|}_{\alpha }+\int_{z_{0}}^{s}{\left\|w(\tau )\right\|}_{\alpha }\;d\tau )\;ds. \end{array}$$ (4)

Where *C*_1_ and *C*_2_ some positive constants depending on *η*, *α*, Ψ_*k*_ and *T*. When the equation is integrating over (*z*_0_, *z*), we have.

$$\begin{array}{c} \int_{z_{0}}^{z}{\left\|w(\xi )\right\|}_{\alpha }\;d\xi \leq C_{1}T+C_{2}\int_{z_{0}}^{z}\int_{z_{0}}^{\xi }{\left(\xi -s\right)}^{\eta -\alpha }({\left\|w(s)\right\|}_{\alpha }+\int_{z_{0}}^{s}{\left\|w(\tau )\right\|}_{\alpha }\;d\tau )\;ds\;d\xi . \end{array}$$

Now for integration changing the order

$$\begin{array}{c} \int_{z_{0}}^{z}{\left\|w(\xi )\right\|}_{\alpha }\;d\xi \leq C_{1}T+C_{2}\int_{z_{0}}^{z}\int_{s}^{z}{\left(\xi -s\right)}^{\eta -\alpha }({\left\|w(s)\right\|}_{\alpha }+\int_{z_{0}}^{s}{\left\|w(\tau )\right\|}_{\alpha }\;d\tau )\;d\xi \;ds. \end{array}$$

We rewrite the equation as

$$\begin{array}{c} \int_{z_{0}}^{z}{\left\|w(\xi )\right\|}_{\alpha }\;d\xi \leq C_{1}T+C_{2}\int_{z_{0}}^{z}(\int_{s}^{z}{\left(\xi -s\right)}^{\eta -\alpha }d\xi )({\left\|w(s)\right\|}_{\alpha }+\int_{z_{0}}^{s}{\left\|w(\tau )\right\|}_{\alpha }\;d\tau )\;ds\\ \leq C_{1}T+\frac{C_{2}T}{\eta -\alpha +1}\int_{z_{0}}^{z}{\left(z-s\right)}^{\eta -\alpha }({\left\|w(s)\right\|}_{\alpha }+\int_{z_{0}}^{s}{\left\|w(\tau )\right\|}_{\alpha }\;d\tau )\;ds. \end{array}$$

The estimate above equation

$$\begin{array}{c} \int_{z_{0}}^{z}{\left\|w(\xi )\right\|}_{\alpha }\;d\xi \leq C_{3}+C_{4}\int_{z_{0}}^{z}{\left(z-s\right)}^{\eta -\alpha }({\left\|w(s)\right\|}_{\alpha }+\int_{z_{0}}^{s}{\left\|w(\tau )\right\|}_{\alpha }\;d\tau )\;ds \end{array}$$ (5)

for some nonnegative *C*_3_ and *C*_4_ depending on *η*, *α*, and *T* only. Adding (4) and (5), we have

$$\begin{array}{c} {\left\|w(z)\right\|}_{\alpha }+\int_{z_{0}}^{z}{\left\|w(\xi )\right\|}_{\alpha }\;d\xi \leq C_{5}+C_{6}\int_{z_{0}}^{z}{\left(z-s\right)}^{\eta -\alpha }({\left\|w(s)\right\|}_{\alpha }+\int_{z_{0}}^{s}{\left\|w(\tau )\right\|}_{\alpha }\;d\tau )\;ds \end{array}$$

for some nonnegative *C*_5_ and *C*_6_ depending on *η*, *α* and *T* only. Using Lemma 4.1 to above we have ‖*w*(*z*)‖ ≤ *C* on [*z*_0_, *T*]. Which is the proof.

## 5 Application

Let ∂Ω be smooth boundary where $\Omega \subseteq R^{n}$ be a bounded. Let the partial differential linear operator

$$\begin{array}{c} A\left(x,D\right)=\sum_{|\alpha |\leq 2m}a_{\alpha }\left(x\right)D^{\alpha }. \end{array}$$

where for each multi-index *α* we defined a real or complex valued map *a*_*α*_(*x*) on $\bar{\Omega }$. Suppose that *A*(*x*, *D*) is strongly elliptic. i.e. there is a positive constant *c* > 0 where

$$\begin{array}{c} \sum_{|\alpha |=2m}a_{\alpha }\left(x\right)\xi^{\alpha }\geq c{\left|\xi \right|}^{2m}. \end{array}$$

for every $x\in \bar{\Omega }$ and $\xi \in R^{n}$. Consider the parabolic IDE.

$$\begin{array}{c} \frac{\partial^{\eta }w\left(x,z\right)}{\partial z^{\eta }}+A\left(x,D\right)w\left(x,z\right)=f(x,z,w\left(x,z\right),Dw\left(x,z\right),...,D^{2m-1}w\left(x,z\right)\\ +\int_{0}^{z}q\left(z-s\right)g\left(x,s,Dw\left(x,z\right),...,D^{2m-1}w\left(x,z\right)\right)\;ds,\;x\in \Omega ,z\geq 0,\\ {\Delta w|}_{z=\frac{1}{2}}=I_{k}(w(x,{\frac{1}{2}}^{-})),\\ w(x,0)+\varphi (w(x,z))=w_{0}\left(x\right),\;x\in \bar{\Omega },\\ w(x,z)=0,\;x\in \bar{\Omega }. \end{array}$$ (6)

Where *D*^*j*^ stands by any jth order derivative. Let *f* and *g* are continuous differentiable functions of all their variable, expect possibly in *x*.

By following abstract IDE in *X* = *L*^*p*^(Ω), we can be reformulated the parabolic IDE (6):

$$\begin{array}{c} \frac{d^{\eta }w\left(z\right)}{dz^{\eta }}+\zeta_{p}w\left(z\right)=F\left(z,w\left(x,z\right)\right)+\int_{0}^{z}q\left(z-s\right)G\left(s,w\left(s\right)\right)\;ds\;z>0\\ {\Delta w|}_{z=\frac{1}{2}}=I_{k}(w({\frac{1}{2}}^{-})),\\ w(0)+\varphi (w(z))=w_{0}. \end{array}$$ (7)

And *ζ*_*p*_: *D*(*ζ*_*p*_) ⊂ *X* → *X* given by

$$\begin{array}{c} D\left(\zeta_{p}\right)=W^{2m,p}\left(\Omega \right)\cap W_{0}^{2m,p}\left(\Omega \right),\;\zeta_{p}w=A\left(x,D\right)w+\lambda w\;\text{for}\;w\in D\left(\zeta_{p}\right),\lambda >0 \end{array}$$

also *F*, *G*: [0, *T*) × *D*(*A*_*p*_) → *X* are the Nemyckii operators is

$$\begin{array}{c} F\left(z,w\right)\left(x\right)=f(x,z,w\left(x,z\right),Dw\left(x,z\right),...,D^{2m-1}w\left(x,z\right)) \end{array}$$ (8)

$$\begin{array}{c} G\left(z,w\right)\left(x\right)=g(x,z,w\left(x,z\right),Dw\left(x,z\right),...,D^{2m-1}w\left(x,z\right)) \end{array}$$ (9)

We can defined the maps *f* and *g* for the Nemyckii operators in (8) and (9) under the assumption sufficient Caratheodory and growth conditions. Now for *λ* is large so that *ζ*_*p*_ in invertible. We have that −*ζ*_*p*_ is IGAS on *X*. And from imbedding theorems we have that *X*_*α*_ is continuous imbedded in $C^{2m-1}\left(\bar{\Omega }\right)$ for 1 − 1/2*m* < *α* < 1 and *p* large value. We verified that *F* and *G* is satisfied by assumption. So a suitable assumptions on the kernel *q*, the existence of a unique global mild solution to (7) is ensures by Theorem 4.1 for *p* large. Hence, the existence of a unique global mild solution to (6) is guarantees.

## 6 Conclusion

In this manuscript, we have successfully established the sufficient conditions for the existence of solutions to fractional equations abstract integro-differential equation with impulsive. Further, we used the Banach fixed point theorem for existence of a unique solution. In the end, example are given to demonstrate the effectiveness of the obtained analytical results. Motivated by this manuscript, we can consider and investigate the existence of fractional order integro-differential equation with impulsive condition on time scales.
