# Correction: Impulsive fractional order integrodifferential equation via fractional operators

**DOI:** 10.1371/journal.pone.0300611

**Published:** 2024-03-11

**Authors:** The PLOS ONE Staff

## Notice of republication

The published article incorrectly associated an incorrect ORCID iD with Ahmad Al-Omari. This article was republished on March 5, 2024 to rectify this error. Please download this article again to view the correct version.
